# The Interferon-Gamma +874 A/T Polymorphism Is Not Associated With CMV Infection After Kidney Transplantation

**DOI:** 10.3389/fimmu.2019.02994

**Published:** 2020-01-08

**Authors:** Jose Luis Santiago, Isabel Pérez-Flores, Luis Sánchez-Pérez, Maria Angeles Moreno de la Higuera, Natividad Calvo-Romero, Javier Querol-García, Esther Culebras, Elena Urcelay, Cristina Fernández-Pérez, Ana Isabel Sánchez-Fructuoso

**Affiliations:** ^1^Hospital Clínico San Carlos, Instituto de Investigación Sanitaria San Carlos (IdISSC), Madrid, Spain; ^2^Nephrology Department Hospital Clínico San Carlos, Facultad de Medicina, Universidad Complutense de Madrid, Instituto de Investigación Sanitaria San Carlos (IdISSC), Madrid, Spain; ^3^Microbiology Department Hospital Clínico San Carlos, Instituto de Investigación Sanitaria San Carlos (IdISSC), Madrid, Spain; ^4^Clinical Research and Methodology Unit, Facultad de Medicina, Hospital Clínico San Carlos Universidad Complutense de Madrid, Instituto de Investigación Sanitaria San Carlos (IdISSC), Madrid, Spain

**Keywords:** cytomegalovirus, IFNG polymorphism, immunosuppression, kidney transplantation, prophylaxis

## Abstract

The +874 A/T polymorphism in the interferon gamma (*IFNG*) gene has been associated with Cytomegalovirus (CMV) infection risk in lung and kidney transplant recipients. To replicate this association, we performed a retrospective observational study of this polymorphism and immunosuppressive therapies considering the prophylactic treatment in 600 consecutive kidney transplanted recipients. We found no association of the aforementioned polymorphism with CMV infection in univariate and multivariate analyses regardless of the prophylactic treatment. In addition, the immunosuppressive treatment with mammalian target of rapamycin inhibitors (imTOR) showed a protective effect in all patients independently of prophylaxis. Moreover, in the adjusted model, we found interactions between prophylaxis with high-risk (Donor+/Recipient–, D+/R–) status (*p*-interaction = 0.01), with thymoglobulin induction therapy (*p*-interaction = 0.03) and with thymoglobulin anti-rejection therapy (*p*-interaction = 0.002). Data also revealed that prophylaxis was not an advantage in the not D+/R– and without thymoglobulin therapy group (HR = 0.98, *p* = 0.95). The benefit of prophylaxis was observed in all groups with thymoglobulin therapy, but it was maximal in the high-risk CMV infection group with both thymoglobulin induction therapy and thymoglobulin anti-rejection therapy (HR = 0.01, *p* < 0.001). In conclusion, the *IFNG* +874 polymorphism is not a predictive marker of CMV infection. The protective effect of imTOR is not improved with prophylaxis. Interestingly, the thymoglobulin therapy associated with prophylaxis is not a risk factor for CMV infection, and prophylaxis is not effective in recipients with no high-risk CMV status and without thymoglobulin therapy.

## Introduction

Cytomegalovirus (CMV) remains one of the most frequent infections affecting organ transplant recipients. It usually appears during the first few months after transplantation (1–3) causing significant morbidity, graft loss, and adverse outcomes (1, 4) even in patients receiving prophylactic treatment for CMV (5). Its clinical manifestations are variable, ranging from asymptomatic viremia to CMV syndrome, the tissue-invasive disease that can lead to allograft injury and to a systemic immunosuppression that predisposes to other opportunistic infections and to malignancy, resulting in a decreased graft and patient survival (6). However, the specific mechanism that could explain the association of CMV infection with diminished graft function is not fully elucidated as there are numerous factors involved (7). First, the serostatus of donor and recipient is a crucial risk factor for the development of CMV disease (2). Second, the immunosuppression, both type and intensity has also a strong influence; an increased incidence of CMV infection has been associated with the use of thymoglobulin (8–12), in contrast to the preventive effect that has been reported regarding treatment with imTOR (13–19). Finally, occurrence of acute rejection is considered an additional risk factor (20); although it is difficult to determine whether allograft rejection is a cause of CMV infection or a consequence of a reduced immunosuppressive therapy in patients with symptomatic CMV disease (5). Toupance et al. have reported an increased risk of graft rejection 1 month after CMV infection (21).

In order to clarify the implications of the virus on transplant outcome, the genetic risk factors of CMV infection have been considered. Thus, some polymorphisms located in genes involved in immune response have been studied (22–25), including the interferon gamma (*IFNG*) gene (26, 27). IFN-γ is a cytokine of the Th1 subset, which has been associated with the inflammatory process and kidney injury, and it plays a critical role in the immune response against viral infection (28–30). The *IFNG* gene is located in chromosome 12q24.1 and the SNP +874 A/T (rs2430561) in the first intron of the gene within the NFkB binding site has been involved in the control of IFN-γ levels (T allele is associated with higher production of IFN-γ) (31, 32). Different genotypes of this SNP have been found associated with increased risk of CMV infection in both, kidney (33) and lung (34) transplant. However, Vu et al. (33) reported association between the AA genotype with increased risk of CMV infection in 247 kidney transplants, while Mitsani et al. (34) reported that the TT genotype, which correlates with high levels of cytokine production, was significantly associated with the development of CMV disease in 170 lung transplants. These apparently controversial results aimed us to replicate the presumed association of the aforementioned polymorphism with CMV infection in a well-powered cohort of 600 kidney recipients.

## Patients and Methods

### Study Design

We performed a retrospective observational study of a kidney transplant cohort. The clinical and research activities being reported are consistent with the *Principles of the Declaration of Helsinki* considering ethical principles for human research. The study was approved by the local ethics committee and written informed consent was obtained from all patients.

### Patients and Clinical Data

Between January 2005 and December 2015, a total of 709 adult patients received a deceased donor organ in our center. We excluded non Caucasian patients, recipients with graft loss during the first month, and patients who died in the immediate postoperative period. A total of 600 patients were studied. All diagnoses of rejection were confirmed by biopsy, and acute rejection was categorized according to the Banff classification (35, 36). Delayed graft function (DGF) was defined as a need for dialysis in the first week after transplant (37).

### Immunosuppression and CMV Prophylaxis

The immunosuppressive protocol varied over time according to physician criteria. Patients who received a kidney from a brain dead donor were treated mainly with tacrolimus, mycophenolate mofetil, and methylprednisolone. When the organ was donated after circulatory death, most patients received treatment with tacrolimus, mycophenolate mofetil, and methylprednisolone combined with basiliximab or thymoglobulin. Thymoglobulin induction therapy refers to the immunosuppressive treatment given with the aim of preventing acute rejection and consisted of 5–7 daily initial doses of 1.25 mg/kg adjusted according to lymphocyte count. In patients who received thymoglobulin, tacrolimus was introduced between days 4 and 6 after transplant.

In our center, prophylaxis is given to all CMV D+/R– patients for 6 months. In all patients treated with thymoglobulin, prophylaxis was maintained for 3 months except in D–/R– patients who did not received prophylaxis. Out of 308 patients with thymoglobulin induction therapy, 276 (89.6%) received prophylaxis. Antiviral prophylaxis started within the first 1–2 weeks after transplant. The antiviral agent used was ganciclovir or valganciclovir depending on whether the estimated glomerular filtration rate (eGFR) was lower or higher than 15 mL/min, respectively, adjusting dose for renal function. The standard prophylaxis with valganciclovir was according to the technical sheet (https://www.rochecanada.com/PMs/Valcyte/Valcyte_PM_E.pdf) and adjusted for estimated CrCl: 900 mg/day when CrCl ≥ 60 mL/ min; 450 mg/day when CrCl = 40–59 mL/min; 450 mg every 2 days when CrCl = 25–39 mL/min; and 450 mg twice a week when CrCl <25 mL/ min.

### Cytokine Polymorphism Genotyping

Genomic DNA was extracted from EDTA-anticoagulated peripheral whole blood. The SNP +874 A/T (rs2430561) was genotyped by TaqMan chemistry and analyzed in a 7900HT Fast Real-Time PCR System (Applied Biosystems, Foster City, CA, USA). The amplification conditions used were those recommended by the manufacturer.

### CMV Infection and Disease

CMV infection was considered as CMV detected in blood in the absence of symptoms. CMV disease was defined according to consensus guidelines on the management of CMV in Solid-Organ transplantation; (20) namely, evidence of CMV infection with attributable symptoms or signs: fever, leucopenia, thrombocytopenia, an increase in transaminases in case of viral syndrome or visceral involvement in case of tissue-invasive disease.

### Diagnosis of CMV

DNA extraction was carried out in the automatic system Eas-MAG (Biomerieux, Madrid Spain) following the manufacturer's instructions. We used Simplexa™ CMV Focus Diagnostic kits to determine CMV viral load by quantitative nucleic acid amplification testing (QNAT). The QNAT was carried out on the 3 M Integrated Cycler Systems with integrated software Cycler Studio version 5.0. The viral load of each sample was expressed in IU/ml, and CMV infection was diagnosed as positive when higher than 3,000 IU/ml.

### Statistical Analysis

Quantitative variables were expressed as mean ± standard deviation (SD) or median with the interquartile range (IQR) for non-normally distributed variables. Association between qualitative variables was evaluated by Chi-square test or Fisher's exact test when necessary. Quantitative variables were compared using the *t*-test or nonparametric tests when necessary. Multivariate Cox's analysis was performed including variables with *p* < 0.15 in the univariate analysis or variables biologically relevant in the population analysis. Interactions with the polymorphisms were also evaluated. The *p* value for the interaction was obtained from the constructed models. Adjusted hazard ratios were presented with their 95% confidence intervals. Statistical analysis used SPSS v. 13.0 software (Chicago, Illinois).

## Results

### Patient Characteristics

The demographic and clinical characteristics of patients with and without CMV infection are summarized in Supplementary Table 1. Out of 600 patients, 205 patients (34.2%) suffered CMV infection and 37 (6.2%) CMV disease. The median time from transplant to the onset of CMV infection was 2.7 months (range 1.2–5.3 months). The incidence of CMV infection was 45.9% in D+/R– patients compared with 10.0% in D–/R– subjects. The mean age of recipients was 52.8 ± 13.4, and 66.3% were male. In the donors group, mean age was 46.1 ± 13.0, and 71.5% were male. Patients with CMV infection were older (*p* < 0.001) and no differences were found for donor age (*p* = 0.47) and gender (*p* = 0.58 for patients and *p* = 0.69 for donors). A total of 354 patients (59.0%) received a kidney from circulatory death donors, 242 (40.3%) after brain deaths, and four patients (0.7%) from a living donor. The incidence of CMV disease in patients that received prophylaxis was: 18.3% in D+/R–, 4.6% in D–/R+, and 2.5% in D+/R+ (*p* < 0.001) and in patients without prophylaxis: 0% in D+/R–, 6.3% in D–/R–, 13.9% in D–/R+, and 4.5% in D+/R+ (*p* = 0.21).

### Impact of *IFNG* +874 A/T Polymorphism on the Incidence of CMV Infection

The genotype distribution of *IFNG* +874 A/T polymorphism in our cohort was: 166 (27.7%) AA, 309 (51.4%) AT, and 125 (20.8%) TT. We also genotyped 536 healthy controls recruited among blood donors from the Madrid area: 152 (28.4%) AA, 275 (51.3%) AT, and 109 (20.3%) TT. Therefore, no statistically significant differences were found between these two groups (OR = 0.98, 95% CI = 0.82–1.16, *p* = 0.87). Both cohorts were under Hardy-Weinberg equilibrium for this polymorphism (*p* = 0.39 and *p* = 0.45 for patients and controls, respectively). The allelic distribution of *IFNG* +874 A/T polymorphism in the patients with CMV infection was no different from recipients without CMV infection (A vs. T, OR = 1.01, 95% CI = 0.79–1.30, *p* = 0.90) nor controls (A vs. T, OR = 0.99, 95% CI = 0.78–1.25, *p* = 0.90).

Cox regression analysis showed no association for genotypes of the *IFNG* +874 A/T polymorphism with CMV infection (Table 1). This lack of association was also found in the groups of patients stratified by prophylactic treatment (Tables 2, 3). Finally, we performed a multivariate analysis and no significant risk was found (Table 4).

**Table 1:** Univariate Cox regression analysis for CMV infection.

	**Probably of CMV-FREE** **Infection at 6 months % (SE)**	**HR (95%CI)**	***p*-value**
***IFNG*** **+874 A/T polymorphism**			
AA (*n* = 166)	72.8 (3.5)	1.09 (0.72–1.65)	0.30
AT (*n* = 309)	71.1 (2.6)	1.30 (0.90–1.87)	0.39
TT (*n* = 125)	74.3 (3.9)	1	0.16
**Donor type**			
Brain death (*n* = 242)	73.3 (2.9)	1	0.35
Circulatory death (*n* = 354)	71.5 (2.4)	1.23 (0.92–1.63)	0.16
Living donors (*n* = 4)	75.0 (21.7)	0.78 (0.11–5.64)	0.81
**Recipient age**			
<60 years (*n* = 388)	75.0 (2.2)	1	
≥60 years (*n* = 212)	67.1 (3.2)	1.41 (1.06–1.86)	0.02
**Prophylaxis**			
Yes (*n* = 391)	78.6 (2.1)	1	
No (*n* = 209)	60.4 (3.4)	1.61 (1.22–2.13)	0.001
**Cold ischemia time**			
≤ 18 h (*n* = 282)	75.1 (2.6)	1	
>18 h (*n* = 318)	69.9 (2.6)	1.31 (1.00–1.73)	0.06
**DGF**			
No (*n* = 316)	76.7 (2.4)	1	
Yes (*n* = 284)	67.2 (2.8)	1.59 (1.21–3.00)	0.001
**Diabetes**			
No (*n* = 530)	73.7 (1.9)	1	
Yes (*n* = 70)	61.1 (5.9)	1.59 (1.10–2.31)	0.01
**High-risk CMV status (D+/R–)**			
No (*n* = 517)	71.9 (2.0)	1	
Yes (*n* = 83)	74.8 (4.8)	1.31 (0.92–1.87)	0.14
**CsA**			
No (*n* = 579)	72.3 (1.9)	1	
Yes (*n* = 21)	71.4 (9.9)	0.84 (0.37–1.89)	0.67
**MMF**			
No (*n* = 21)	71.4 (9.9)	1	
Yes (*n* = 579)	72.3 (1.9)	1.19 (0.53–2.68)	0.67
**imTOR** ***de novo***			
No (*n* = 583)	71.6 (1.9)	1	
Yes (*n* = 17)	94.1 (5.7)	0.14 (0.02–0.09)	0.05
**Tacrolimus**			
No (*n* = 27)	70.4 (8.8)	1	
Yes (*n* = 573)	72.7 (1.9)	1.14 (0.56–2.32)	0.71
**Basiliximab**			
No (*n* = 412)	76.3 (2.1)	1	
Yes (*n* = 188)	63.5 (3.5)	1.66 (1.26–2.20)	<0.001
**Thymoglobulin induction therapy**			
No (*n* = 292)	63.5 (2.8)	1	
Yes (*n* = 308)	80.6 (2.3)	0.54 (0.41–0.72)	<0.001
**Thymoglobulin anti-rejection therapy**			
No (*n* = 489)	75.1 (2.0)	1	
Yes (*n* = 111)	59.4 (4.7)	1.96 (1.44–2.68)	<0.001

*CI, confidence interval; CsA, cyclosporin A; DGF, delayed graft function; HR, hazard ratio; imTOR, mammalian target of rapamycin inhibitors; MMF, mycophenolate; SE, standard error*.

**Table 2:** Univariate Cox regression analysis for CMV infection in patients with prophylaxis.

	**Probably of CMV-FREE** **Infection at 6 months % (SE)**	**HR (95% CI)**	***p*-value**
***IFNG*** **+874 A/T polymorphism**			
AA (*n* = 108)	77.8 (4.0)	1.23 (0.71–2.14)	0.17
AT (*n* = 197)	77.3 (3.0)	1.56 (0.96–2.54)	0.47
TT (*n* = 86)	82.4 (4.1)	1	0.08
**Donor type**			
Brain death (*n* = 127)	79.3 (3.6)	1	0.67
Circulatory death (*n* = 262)	78.1 (2.6)	1.20 (0.81–1.70)	0.37
Living donors (*n* = 2)	100	NA	0.95
**Recipient age**			
<60 years (*n* = 257)	79.9 (2.5)	1	
≥60 years (*n* = 134)	75.9 (3.7)	1.33 (0.93–1.92)	0.12
**Cold ischemia time**			
≤ 18 h (*n* = 197)	79.1 (2.9)	1	
>18 h (*n* = 194)	78.4 (3.0)	1.21 (0.84–1.73)	0.31
**DGF**			
No (*n* = 183)	80.1 (3.0)	1	
Yes (*n* = 208)	77.2 (2.9)	1.25 (0.87–1.74)	0.23
**Diabetes**			
No (*n* = 345)	79.8 (2.2)	1	
Yes (*n* = 46)	69.5 (6.8)	1.63 (1.01–2.63)	0.05
**High-risk CMV status (D+/R–)**			
No (*n* = 309)	79.4 (2.3)	1	
Yes (*n* = 82)	75.4 (4.8)	1.69 (1.14–2.49)	0.009
**CsA**			
No (*n* = 384)	78.4 (2.1)	1	
Yes (*n* = 7)	85.7 (13.8)	0.43 (0.06–3.07)	0.40
**MMF**			
No (*n* = 7)	85.7 (13.2)	1	
Yes (*n* = 384)	78.4 (2.1)	2.33 (0.33–16.68)	0.40
**imTOR** ***de novo***			
No (*n* = 379)	78.1 (2.1)	1	
Yes (*n* = 12)	91.7 (8.0)	0.22 (0.03–1.59)	0.13
**Tacrolimus**			
No (*n* = 11)	81.8 (11.6)	1	
Yes (*n* = 380)	78.5 (2.1)	1.82 (0.45–7.37)	0.40
**Basiliximab**			
No (*n* = 315)	81.4 (2.2)	1	
Yes (*n* = 76)	66.7 (5.5)	2.11 (1.43–3.13)	<0.001
**Thymoglobulin induction therapy**			
No (*n* = 115)	66.7 (2.2)	1	
Yes (*n* = 276)	83.5 (4.4)	0.46 (0.32–0.66)	<0.001
**Thymoglobulin anti-rejection therapy**			
No (*n* = 314)	79.9 (2.3)	1	
Yes (*n* = 77)	73.2 (5.1)	1.47 (0.97–2.23)	0.07

*CI, confidence interval; CsA, cyclosporin A; DGF, delayed graft function; HR, hazard ratio; imTOR, mammalian target of rapamycin inhibitors; MMF, mycophenolate; SE, standard error*.

**Table 3:** Univariate Cox regression analysis for CMV infection in patients without prophylaxis.

	**Probably of CMV-free** **infection at 6 months % (SE)**	**HR (95%CI)**	***p*-value**
***IFNG*** **+874 A/T polymorphism**			
AA (*n* = 58)	63.8 (6.3)	0.86 (0.46–1.62)	0.88
AT (*n* = 112)	69.4 (1.8)	0.95 (0.55–1.67)	0.64
TT (*n* = 39)	56.4 (7.9)	1	0.87
**Donor type**			
Brain death (*n* = 115)	66.7 (4.4)	1	0.09
Circulatory death (*n* = 92)	52.8 (5.2)	1.63 (1.06–2.51)	0.03
Living donors (*n* = 2)	50.0 (35.4)	1.44 (0.19–10.53)	0.72
**Recipient age**			
<60 years (*n* = 131)	65.4 (4.2)	1	
≥60 (*n* = 78)	52.0 (5.7)	1.39 (0.90–2.14)	0.14
**Cold ischemia time**			
≤ 18 h (*n* = 85)	65.9 (5.1)	1	
>18 h (*n* = 124)	56.6 (4.5)	1.36 (0.87–2.12)	0.18
**DGF**			
No (*n* = 133)	72.0 (3.9)	1	
Yes (*n* = 76)	39.9 (5.7)	2.88 (1.87–4.43)	<0.001
**Diabetes**			
No (*n* = 185)	62.4 (3.6)	1	
Yes (*n* = 24)	45.8 (10.2)	1.42 (0.79–2.57)	0.24
**High-risk CMV status (D+/R–)**			
No (*n* = 208)	60.7 (3.4)	1	
Yes (*n* = 1)	0	8.94 (1.21–66.37)	0.03
**CsA**			
No (*n* = 195)	60.1 (3.5)	1	
Yes (*n* = 14)	64.3 (12.8)	0.82 (0.33–2.03)	0.67
**MMF**			
No (*n* = 14)	64.3 (12.8)	1	
Yes (*n* = 195)	59.6 (3.5)	1.22 (0.49–3.00)	0.67
**imTOR** ***de novo***			
No (*n* = 204)	59.4 (3.5)	1	
Yes (*n* = 5)	100	0.05 (0.00–11.76)	0.28
**Tacrolimus**			
No (*n* = 16)	62.5 (12.1)	1	
Yes (*n* = 193)	60.2 (3.5)	1.11 (0.48–2.04)	0.81
**Basiliximab**
No (*n* = 97)	59.4 (5.0)	1	
Yes (*n* = 112)	61.3 (4.6)	1.00 (0.65–1.54)	1.00
**Thymoglobulin induction therapy**			
No (*n* = 177)	61.4 (3.7)	1	
Yes (*n* = 32)	54.8 (9.0)	1.19 (0.67–2.11)	0.55
**Thymoglobulin anti-rejection therapy**			
No (*n* = 175)	66.5 (3.6)	1	
Yes (*n* = 34)	28.9 (7.9)	3.46 (2.16–5.55)	<0.001

*CI, confidence interval; CsA, cyclosporin A; DGF, delayed graft function; HR, hazard ratio; imTOR, mammalian target of rapamycin inhibitors; MMF, mycophenolate; SE, standard error*.

**Table 4:** Multivariate Cox regression analysis of CMV infection.

**Risk factor**	**HR**	**95%CI**	***p*-value**
***IFNG*** **+874 A/T polymorphism**			
AA	1.02	0.67–1.56	0.52
AT	1.19	0.82–1.72	0.93
TT	1		0.36
Recipient age ≥60 years	1.49	1.12–1.99	0.006
Diabetes	1.27	0.86–1.87	0.22
Cold ischemia time >18 h	1.20	0.90–1.60	0.22
DGF	1.73	1.28–2.33	<0.001
imTOR *de novo*	0.15	0.02–1.08	0.06
Basiliximab	0.90	0.60–1.34	0.60
HR values of prophylaxis vs. no prophylaxis^*^			
1. High-risk CMV status (D+/R–)			
a. Thymoglobulin induction therapy			
i. Thymoglobulin anti-rejection therapy	0.01	0.00–0.08	<0.001
ii. No Thymoglobulin anti-rejection therapy	0.03	0.00–0.21	0.001
b. No Thymoglobulin induction therapy			
i. Thymoglobulin anti-rejection therapy	0.02	0.00–0.19	0.001
ii. No Thymoglobulin anti-rejection therapy	0.06	0.01–0.51	0.01
2. No high-risk CMV status			
a. Thymoglobulin induction therapy			
i. Thymoglobulin anti-rejection therapy	0.15	0.07–0.33	<0.001
ii. No Thymoglobulin anti-rejection therapy	0.43	0.23–0.82	0.01
b. No Thymoglobulin induction therapy			
i. Thymoglobulin anti-rejection therapy	0.34	0.19–0.61	<0.001
ii. No Thymoglobulin anti-rejection therapy	0.98	0.58–1.67	0.95

*CI, confidence interval; DGF, Delayed graft function; HR, hazard ratio; imTOR, mammalian target of rapamycin inhibitors*.

* p-interaction of prophylaxis with:

*High-risk CMV status p-interaction = 0.010*.

*Thymoglobulin induction therapy p-interaction = 0.034*.

*Thymoglobulin anti-rejection therapy p-interaction = 0.002*.

Kaplan-Meier curves for CMV infection-free survival according to *IFNG* +874 A/T genotypes evidenced no differences in patients receiving prophylaxis or not (Supplementary Figure 1).

In CMV disease, the genotype distribution of the *IFNG* +874 A/T polymorphism was not different (*p* = 0.58) between patients who developed CMV disease (*n* = 37: 12 AA (32.4%), 16 (43.2%) AT, 9 (24.3%) TT and those who did not (*n* = 583: 154 (27.4%) AA, 293 (52.0%) AT, 116 (20.6%) TT.

Finally, we decided to analyze the association of *IFNG* +874 A/T polymorphisms with allograft rejection, and no associations were found (*p* = 0.90 for acute cellular rejection and *p* = 0.52 for humoral rejection).

### Effect of Immunosuppressive Treatment and Clinical Factors on the Risk of CMV Infection in Kidney Transplant Recipients Receiving or Not Prophylaxis

In univariate analysis, we studied the incidence of CMV infection in all patients (Table 1) and in the groups stratified by prophylaxis (Tables 2, 3, respectively). In Table 1, the recipient age, no prophylaxis, DGF, diabetes, basiliximab and thymoglobulin anti-rejection therapy were all risk factors for CMV infection; imTOR *de novo* and thymoglobulin induction therapy showed a protective effect. In the prophylaxis group (Table 2), only basiliximab and thymoglobulin anti-rejection therapy remained as risk factors, as well as high-risk CMV status. The protective effect was only observed for thymoglobulin induction therapy. Finally, in the group of recipients without prophylaxis (Table 3) DGF, high-risk CMV status and thymoglobulin anti-rejection therapy appeared as risk factors.

When we performed the multivariate analysis, only recipient age and DGF were clinical factors significantly associated with increased risk of CMV infection; and for immunotherapy, only the protective effect of imTOR was close to significance (Table 4). We have also observed interactions between the high-risk CMV status (D+/R–), thymoglobulin induction therapy, and thymoglobulin anti-rejection therapy in groups of patients with prophylaxis vs. no prophylaxis. However, the no-high-risk CMV status patients without thymoglobulin therapy (neither induction nor anti-rejection), did not benefit from prophylaxis (Table 4).

Finally, in patients with thymoglobulin anti-rejection therapy and also in subjects with thymoglobulin induction therapy, the CMV infection-free survival was significantly reduced in no prophylaxis vs. prophylaxis, as shown by Kaplan-Meir curves (Supplementary Figures 2A, 3A). However, in patients without thymoglobulin therapy no differences were found between prophylaxis vs. no prophylaxis (Supplementary Figures 2B, 3B).

We also studied the influence of the *IFNG* +874 A/T polymorphism on the development of CMV infection in the subgroups stratified by thymoglobulin therapy, and we found no association in any of the tested groups: in the overall cohort, in the thymoglobulin induction therapy, in the thymoglobulin anti-rejection therapy, and also in the no thymoglobulin therapy group (Supplementary Table 2).

## Discussion

Cytomegalovirus (CMV) is one of the most common opportunistic viral infections in kidney transplant recipients (38). The infection usually develops few months after transplantation and ranges from asymptomatic to CMV syndrome. In addition to these direct effects, CMV infection predisposes to opportunistic infections and malignancy leading to allograft loss (39) and also to reduced patient survival (6). The incidence in patients receiving prophylaxis within 1 year varies depending on the infective status of both donor and recipient, ranging from 73% in D+/R– to 24% in D–/R+ (40). Options for CMV prevention include prophylaxis with antiviral therapy (valganciclovir is nowadays the standard of care), although it is associated with side effects and unfortunately does not completely prevent virus reactivation; in fact, relapses after cessation of the drug are frequent (41). Other molecules which actively interfere in viral replication are some cytokines such as IFN-γ (42); serum levels of this cytokine have been found elevated during primary CMV infection (30).

In order to predict risk of CMV infection, the *IFNG* +874 A/T polymorphism that affects the level of this cytokine has been evaluated. Related to solid-organ transplantation, Vu et al. (33) in kidney transplant and Mitsani et al. (34) in lung transplant have previously reported the association of different genotypes in this polymorphism with increased risk of CMV infection.

Due to the antiviral role of this cytokine and the implication of the polymorphism modulating its level, we pursued to assess its role on the risk of CMV infection; however, this association was not replicated in our cohort. No differences in the overall incidence of CMV infection were observed according to the genotype of *IFNG* +874 A/T SNP (Table 1). This lack of association was independent of prophylaxis (Tables 2, 3); in fact, in the multivariate analysis none of the studied groups showed association (Table 4). Moreover, in terms of CMV-free allograft survival considering the prophylaxis, a Kaplan-Meier analysis did not evidence an effect as a risk factor of any genotype of the *IFNG* +874 A/T polymorphism (Supplementary Figure 1). When we stratified our cohort by thymoglobulin therapy and analyze the association of this polymorphism with CMV infection, we found no significant differences in any of the tested groups: with any type of thymoglobulin therapy, with thymoglobulin induction therapy, with thymoglobulin anti-rejection therapy and without thymoglobulin therapy (Supplementary Table 2).

Our study is the first attempt to confirm, in a large number of patients (600 consecutive kidney transplanted recipients, 205 patients with CMV infection), the previously reported association of *IFNG* +874 A/T polymorphism with CMV infection in kidney transplant (33) (247 patients, 52 with CMV infection) and in lung transplant (34) (170 recipients, 40 with CMV infection). The effect was not ratified in our cohort; however, some well-established risk factors for CMV infection, as high-risk CMV status, recipient age, DGF, and prophylaxis showed the expected association, indicative of the statistical power of our study (Table 4). The lack of association of this polymorphism with allograft rejection (either acute cellular rejection or humoral rejection) was also evidenced.

On the other hand, it has been widely reported that the use of thymoglobulin as either induction or anti-rejection therapy is associated with high risk of CMV infection (8–12). In fact, the risk is maximal in the thymoglobulin treatment of acute rejection, as the occurrence of acute rejection is a risk factor for infection by itself (20). However; our data showed a protective effect of thymoglobulin induction therapy, which can be explained considering that almost all patients under this treatment received prophylaxis (89.6%). The prophylaxis was beneficial even to patients with the higher risk for CMV infection (D+/R–) under thymoglobulin therapy, for both induction and anti-rejection or only one of them (Table 4). In the group of no-high-risk CMV status, prophylaxis was also protective with thymoglobulin therapy (Table 4), probably due to the fact that thymoglobulin induces lymphocyte depletion and prophylaxis would be more effective in these patients. Moreover, in patients with no-high-risk CMV status and without thymoglobulin therapy, prophylaxis would not provide a benefit, since they have not lymphocyte depletion and have the lowest risk for CMV infection (HR = 0.98, *p* = 0.95, Table 4). As shown in these patients, prophylaxis only delayed the onset of CMV infection, and after 6 months the incidence was similar (Supplementary Figure 3B).

Finally, the previously reported antiviral effect of imTOR (13–19) was found in the univariate analysis in the whole population, and in the multivariate analysis appeared close to significance as an independent factor (Table 4). In the stratified groups, we found no association probably because of a reduced statistical power; nevertheless, in both groups the HR was below one, and it was even lower in the group of recipients without prophylaxis. This seems to support the idea that prophylaxis does not offer a benefit in patients with imTOR treatment.

## Conclusion

The previously published predictive role of *IFNG* +874 A/T polymorphism as a biomarker for increased risk of CMV infection was not replicated in a statistically well-powered Spanish cohort of kidney transplant patients. Thymoglobulin induction therapy does not increase the risk of CMV infection when it is associated with prophylaxis; prophylaxis does not reduce the risk in the no D+/R– group of recipients without thymoglobulin treatment; and the protective effect of imTOR is not improved with the prophylactic treatment.

## Data Availability Statement

The raw data supporting the conclusions of this article will be made available by the authors, without undue reservation, to any qualified researcher. Data are available under accession number PRJEB35786.

## Ethics Statement

The studies involving human participants were reviewed and approved by CEIC Hospital Clínico San Carlos. The patients/participants provided their written informed consent to participate in this study.

## Author Contributions

JS and AS-F designed the study. IP-F, MM, and NC-R were responsible for the clinical care of the patients. JS drafted the manuscript. EU and AS-F revised the manuscript. LS-P and JQ-G performed the data collection. EC performed the virus analysis. CF-P contributed to the data interpretation and critical revision. All authors approved the final version of the manuscript and agreed to submit it for publication.

### Conflict of Interest

The authors declare that the research was conducted in the absence of any commercial or financial relationships that could be construed as a potential conflict of interest.

## Supplementary Material

Supplementary Figure 1 Kaplan–Meier CMV infection-free survival curves according to genotypes of the IFN-γ +874 A/T polymorphism. **(A)** In patients with prophylactic therapy. **(B)** In patients without prophylactic therapy. *P* according to log-rank test.

Supplementary Figure 2 Kaplan–Meier CMV infection-free survival curves comparing the influence of prophylaxis vs. no prophylaxis during 1 year follow-up. **(A)** In patients with thymoglobulin anti-rejection therapy. **(B)** In patients without thymoglobulin anti-rejection therapy. *P* according to log-rank test.

Supplementary Figure 3 Kaplan–Meier CMV infection-free survival curves comparing the influence of prophylaxis vs. no prophylaxis during 1 year follow-up. **(A)** In patients with thymoglobulin induction therapy. **(B)** In patients without thymoglobulin induction therapy. *P* according to log-rank test.

Supplementary Table 1 Demographic and clinic characteristics of the kidney transplant cohort.

Supplementary Table 2 Univariate analysis for CMV infection by the thymoglobulin therapy.
