# Supplementary figures and images for: The Interferon-Gamma +874 A/T Polymorphism Is Not Associated With CMV Infection After Kidney Transplantation
Source: Front Immunol. 2020 Jan 8;10:2994. doi: 10.3389/fimmu.2019.02994 (PMC6961530; doi:10.3389/fimmu.2019.02994)

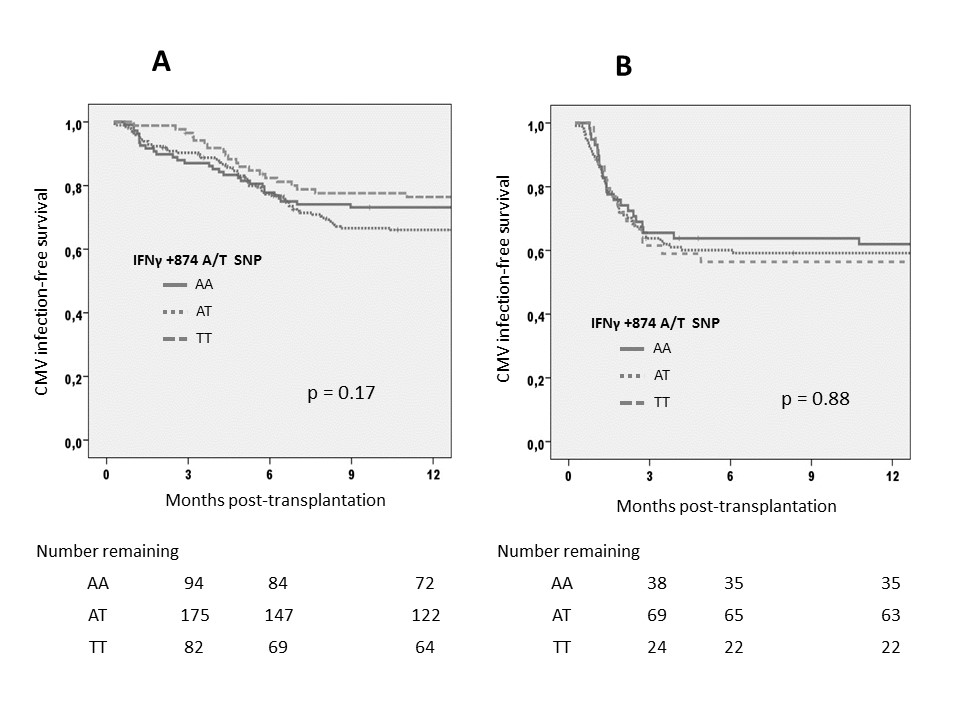

Supplement: Supplementary Figure 1 — Kaplan–Meier CMV infection-free survival curves according to genotypes of the IFN-γ +874 A/T polymorphism. (A) In patients with prophylactic therapy. (B) In patients without prophylactic therapy. P according to log-rank test. [file Image_1.JPEG]

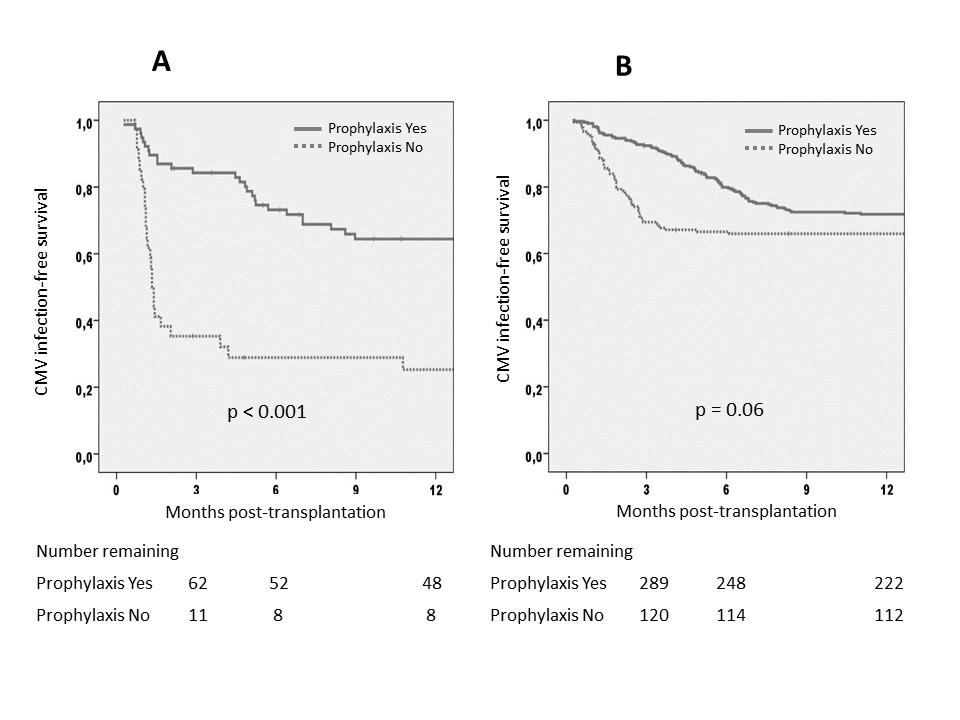

Supplement: Supplementary Figure 2 — Kaplan–Meier CMV infection-free survival curves comparing the influence of prophylaxis vs. no prophylaxis during 1 year follow-up. (A) In patients with thymoglobulin anti-rejection therapy. (B) In patients without thymoglobulin anti-rejection therapy. P according to log-rank test. [file Image_2.JPEG]

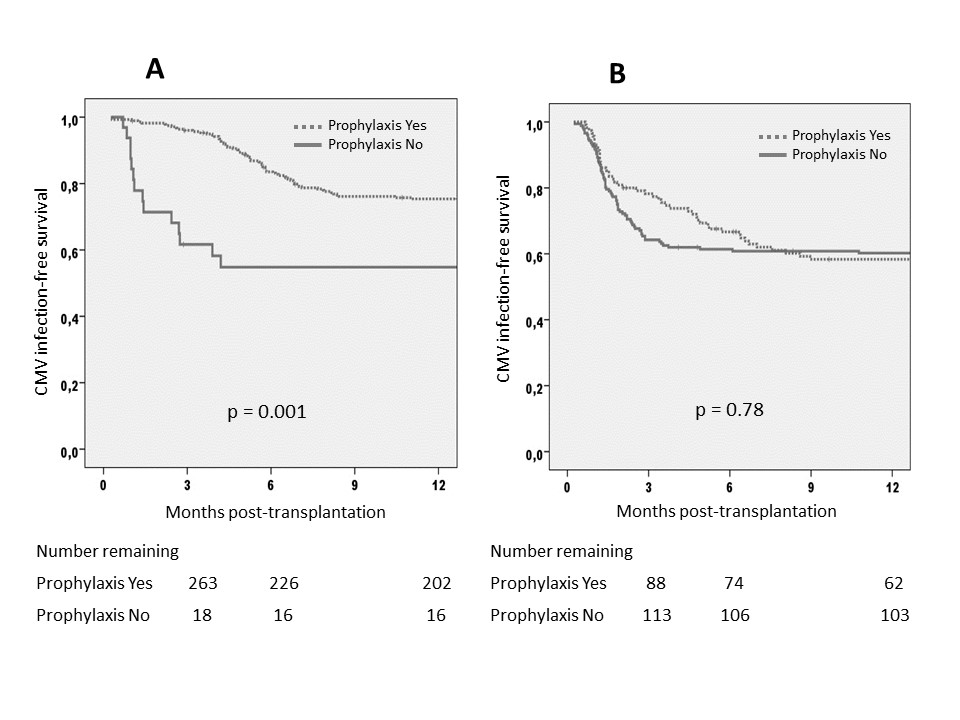

Supplement: Supplementary Figure 3 — Kaplan–Meier CMV infection-free survival curves comparing the influence of prophylaxis vs. no prophylaxis during 1 year follow-up. (A) In patients with thymoglobulin induction therapy. (B) In patients without thymoglobulin induction therapy. P according to log-rank test. [file Image_3.JPEG]
