# Supplementary material for: The Interferon-Gamma +874 A/T Polymorphism Is Not Associated With CMV Infection After Kidney Transplantation
Source: Front Immunol. 2020 Jan 8;10:2994. doi: 10.3389/fimmu.2019.02994 (PMC6961530; doi:10.3389/fimmu.2019.02994)
Supplement: Supplementary Table 1 — Demographic and clinic characteristics of the kidney transplant cohort. [file Table_1.DOCX]

**Supplementary Table 1** Demographic and clinic characteristics of the kidney transplant cohort.

| Characteristic | No CMV infection (n=395) | CMV infection  (n=205) | p-value |
| --- | --- | --- | --- |
| Recipient age, years, mean ± SD | 51.3 ± 13.4 | 55.5 ± 12.9 | <0.001 |
| Male recipient, N (%) | 259 (65.6%) | 139 (67.8%) | 0.58 |
| Follow-up time, months, Median (IQR) | 51.7 (31.2-83.7) | 66.4 (40.4-94.3) | 0.002 |
| Cause of Chronic renal failure, N (%)  Glomerulonephritis  Chronic tubulointerstitial Nephropathy  [Nephroangiosclerosis](https://www.ncbi.nlm.nih.gov/pubmed?term=nephroangiosclerosis&cmd=correctspelling)  Polycystic kidney disease  Diabetic nephropathy  Unknown cause  Others | 144 (36.5%)  64 (16.2%)  17 (4.3%)  60 (15.2%)  37 (9.4%)  55 (13.9%)  18 (4.6%) | 57 (27.8%)  25 (12.2%)  15 (7.3%)  32 (15.6%)  32 (15.6%)  35 (17.1%)  9 (4.4%) | 0.05 |
| Donor age, years, mean ± SD | 46.3 ± 12.5 | 45.5 ± 13.9 | 0.47 |
| Male donor, N (%) | 279 (70.6%) | 148 (72.2%) | 0.69 |
| Donor type, N (%)  Brain death  Circulatory death  Living donors | 169 (42.8%)  223 (56.5%)  3 (0.8%) | 73 (35.6%)  131 (63.9%)  1 (0.5%) | 0.25 |
| Initial immunosuppressive treatment, N (%)  CsA  MMF  imTOR  Tacrolimus  Basiliximab | 15 (3.8%)  380 (96.2%)  16 (4.1%)  376 (95.2%)  108 (27.3%) | 6 (2.9%)  199 (97.1%)  1 (0.5%)  197 (96.1%)  80(39.0%) | 0.58  0.58  0.01  0.61  0.003 |
| Thymoglobulin induction therapy, N (%) | 224 (56.7%) | 84 (41.0%) | <0.001 |
| Thymoglobulin anti-rejection therapy, N (%) | 151 (38.2%) | 54 (26.3%) | <0.001 |
| Prophylaxis, N (%) | 270 (68.4%) | 121 (59.0%) | 0.032 |
| Recipient with CMV infection, N (%) | 331 (83.8%) | 165 (80.5%) | 0.31 |
| Donor with CMV infection, N (%) | 306 (77.5%) | 172 (83.9%) | 0.06 |
| DGF, N (%) | 168 (42.5%) | 116 (56.6%) | 0.001 |
| HLA-A mismatch, mean ± SD | 1.4 ± 0.65 | 1.4 ± 0.63 | 0.93 |
| HLA-B mismatch, mean ± SD | 1.5 ± 0.57 | 1.5 ± 0.56 | 0.69 |
| HLA-DR mismatch, mean ± SD | 1.3 ± 0.64 | 1.3 ± 0.65 | 0.81 |
| Graft loss, N (%) | 38 (9.6%) | 25 (12.2%) | 0.34 |
| Death, N (%) | 34 (8.6%) | 20 (9.8%) | 0.66 |

CsA, cyclosporin A; DGF, delayed graft function; IQR, interquartile range; imTOR, mammalian target of rapamycin inhibitors; MMF, mycophenolate; SD, standard deviation
