# Supplementary material for: The Interferon-Gamma +874 A/T Polymorphism Is Not Associated With CMV Infection After Kidney Transplantation
Source: Front Immunol. 2020 Jan 8;10:2994. doi: 10.3389/fimmu.2019.02994 (PMC6961530; doi:10.3389/fimmu.2019.02994)
Supplement: Supplementary Table 2 — Univariate analysis for CMV infection by the thymoglobulin therapy. [file Table_2.DOCX]

**Supplementary Table 2** Univariate analysis for CMV infection by the thymoglobulin therapy.

|  | CMV infection  N (%) | No CMV infection  N (%) | p-value |
| --- | --- | --- | --- |
| **Thymoglobulin therapy (overall)**  *IFNG*+874 A/T polymorphism  AA  AT  TT | 71 (71.7)  126 (63.6)  59 (68.6) | 28 (28.3)  72 (36.4)  27 (31.4) | 0.350 |
| **Thymoglobulin induction therapy**  *IFNG*+874 A/T polymorphism  AA  AT  TT | 61 (77.2)  110 (70.1)  53 (73.6) | 18 (22.8)  47 (29.9)  19 (26.4) | 0.499 |
| **Thymoglobulin anti-rejection therapy**  *IFNG*+874 A/T polymorphism  AA  AT  TT | 14 (51.9)  34 (53.1)  9 (45.0) | 13 (48.1)  30 (46.9)  11 (55.0) | 0.816 |
| **No Thymoglobulin therapy**  *IFNG*+874 A/T polymorphism  AA  AT  TT | 42 (62.4)  69 (62.2)  28 (71.8) | 25 (37.3)  42 (37.8)  11 (28.2) | 0.533 |
